# First record of larvae of the water mite *Hydrachna processifera* Piersig, 1895 from Turkey (Acari, Hydrachnidia, Hydrachnidae)

**DOI:** 10.3897/zookeys.738.21021

**Published:** 2018-02-19

**Authors:** Medeni Aykut, Andrzej Zawal, Yunus Esen, Orhan Erman

**Affiliations:** 1 Dicle University, Ziya Gökalp Education Faculty, Department of Mathematics and Science, Diyarbakır, Turkey; 2 University of Szczecin, Faculty of Biology, Institute for Research on Biodiversity, Department of Invertebrate Zoology & Limnology, Centre of Molecular Biology & Biotechnology, 71 – 415 Szczecin, Poland; 3 Bingol University, Solhan Vocational School of Health Services, Medical Services and Techniques, Bingöl, Turkey; 4 Fırat University, Faculty of Science, Elazığ, Turkey

**Keywords:** *Dytiscus marginalis*, *Hydrachna inermis*, parasitism, water beetles, water mites

## Abstract

Larvae of water mite *Hydrachna processifera* Piersig, 1895 (Acari, Hydrachnidiae) were reported on diving beetles *Dytiscus marginalis* Linnaeus, 1758 (Coleoptera, Dytiscidae) from Turkey. The redescription of the larva was made. Earlier, the larva *H. processifera* was described as *H. inermis*, but it was subsequently synonymized with *H. processifera*. The larva of *H. processifera* is a new record for the Turkish fauna. All larvae of *H. processifera* were found on the mesosternum of the one specimens (prevalence = 16.7%).

## Introduction

Mite taxonomy issues continue to pose some difficulties, which causes many synonymic names of particular species. The situation is much more difficult because of the presence of pre-adult stages such as larvae and deutonymphs. For instance, *Hydrachna inermis* Piersig, 1895 has been synonymized with *H. processifera* Piersig, 1895 by Davids et al. (2005, 2007). Larvae of *H. inermis* were described by Sparing (1959) and Wainstein (1980) based on variable and questionable features of adults and these larvae were synonymized with *H. processifera* as well (Davids et al. 2005, 2007). All subsequent information about the parasitic behavior of *H. inermis* on Dytiscidae (Sparing 1959, Zawal 2002) should be recognized as *H. processifera*.

Larvae of water mites of the genera *Hydrachna*, *Eylais*, *Limnochares*, and *Acherontacarus* are ectoparasites on aquatic Hemiptera and aquatic Coleoptera (Reilly and McCarthy 1993, Biesiadka and Cichocka 1994, Cichocka 1995, Benfatti and Gerecke 1999, Zawal 2002, 2003a, 2003b, Ihle and McCreadie 2003, Fairn et al. 2008, Zawal et al. 2013, Aykut et al. 2016, Aykut and Esen 2017).

Parasitizing larvae of *H. processifera* (as *H. inermis*) were reported on Dytiscidae and Hydrophilidae in previous studies (Piatakov 1915a, 1915b, Brumpt 1929, Davids 1969, Zawal 2002). Zawal (2002) reported that *H. inermis* occurred as the most frequent parasites of *Dytiscus* (*D. circumcinctus* (Ahrens, 1811), *D. dimidiatus* Bergsträsser, 1778, and *D. marginalis* Linnaeus, 1758). In Turkey, studies on larvae of water mite are not advanced and only several studies were published (İncekara and Erman 2008, Taşar et al. 2012, Zawal et al. 2013, Aykut et al. 2016, Aykut and Esen 2017). In Turkey only six species of the genus *Hydrachna* (*H. conjecta* (Koenike, 1895), *H. globosa* (De Geer, 1778), *H. leegei* (Koenike, 1895), *H. orientalis* (Thon, 1905), *H. processifera* (Koenike, 1903), and *H. skorikowi* (Piersig, 1900)) were previously known (Erman et al. 2010). This study contributes to larval morphology of *H. processifera* and its parasitization on *Dytiscus marginalis*.

## Materials and methods

**Figure 1.**
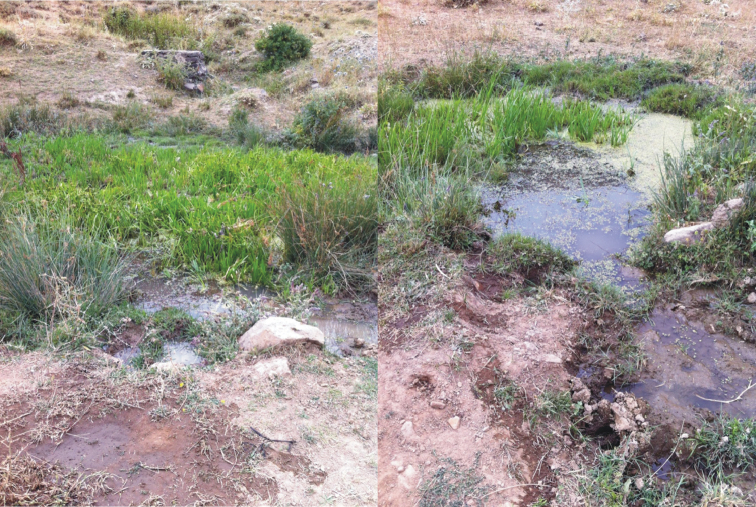
Collecting site of *Dytiscus marginalis* infected with *Hydrachna processifera* larvae.

Parasitized specimens of *Dytiscus marginalis* were collected from a small pond supplied by a small water source in the plateau near Çayıryolu village of Varto district (39°09'23"N, 41°34'56"E; 20.08.2014) in the Eastern Anatolia Region of Turkey (Fig. 1). The coordinates and altitude information of the locality were taken directly from a handheld GPS tool (Magellan Explorist 610). The beetles were collected with a net of mesh size 0.5 mm diameter. Specimens were fixed and preserved in 70% ethyl alcohol solution at the collection site. The clay and muddy substance on their surfaces was brushed off with a small paint brush in the laboratory and each specimen was checked for the presence of water mites under a stereomicroscope. Beetle species were identified according to Friday (1988), Nilsson and Holmen (1995), Pederzani (1995), and Nilsson (1996) and mite larvae according to Wainstein (1980). Photographs were taken using stereo microscope (Z16 APO; Leica, Wetzlar, Germany) equipped with an HD camera (Leica MC170), and with a scanning electron microscope (Quanta 250 FEG; FEI, Eindhoven, Netherlands). The examined material is deposited in the private collection of the first author, at Dicle University, Diyarbakır, Turkey.

The following abbreviations are used: **Cx** – coxa; **L** – length; **W** – width.

## Results

In total, six specimens of *D. marginalis* including four females and two males were collected. Of these, only one female specimen was infected (prevalence = 16.7%). Except *D. marginalis*, a total of 37 specimens belongs to four genera and seven species (*Agabus biguttatus*, *A. bipustulatus*, *A. conspersus*, *Colymbetes fuscus*, *Hydroporus pubescens*, *H. tesellatus*, *Platambus maculatus*). None of them was positive with regards to water mites.

**Figure 2.**
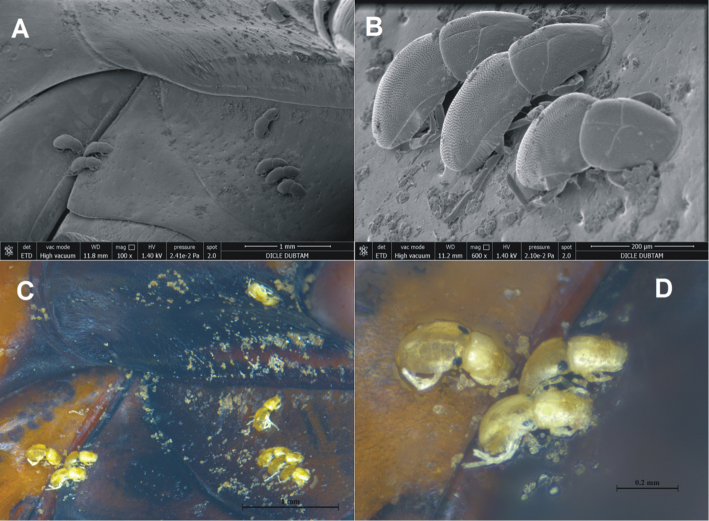
SEM (**A, B**) and stereomicroscope (**C, D**) images of *Dytiscus marginalis* infected with larvae of water mites *Hydrachna processifera*.

**Figure 3.**
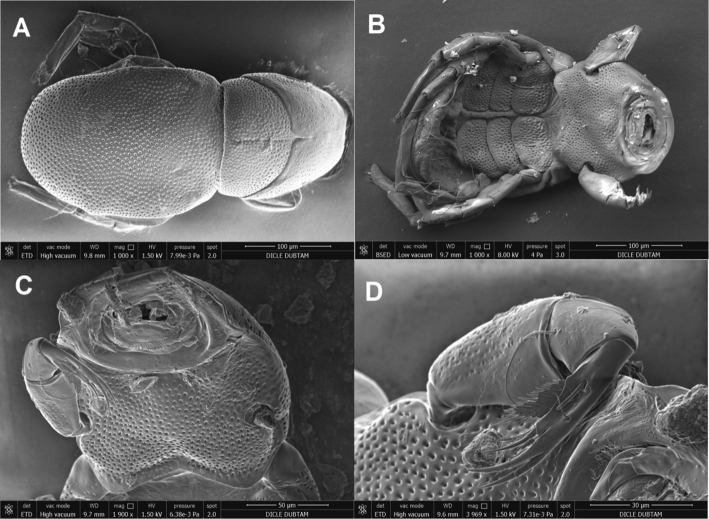
SEM images of larvae of *Hydrachna processifera*. **A** dorsal view **B** ventral view **C** ventral view of gnatosoma **D** lateral view of palp.

**Figure 4.**
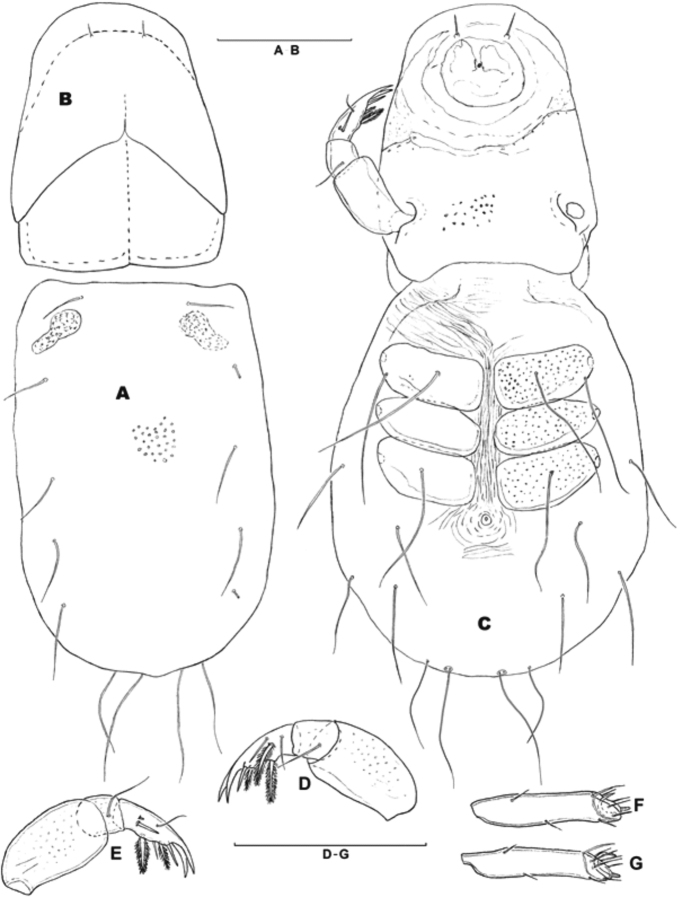
Larvae of *Hydrachna processifera*: **A** dorsal shield **B** dorsal view of gnatosoma **C** ventral view **D** palp, medial view **E** palp, lateral view **F** I leg, tarsus **G** II leg, tarsus. Scale bars 100 µm.

On *D. marginalis*, eleven larvae of *H. processifera* were observed (Fig. 2). All larvae were found on the surface of mesosternum of the beetle body and they were small, 0.15–0.40 mm. The idiosoma are egg-shaped, with the integument striated, and the dorsal shield is very large, covered whole idiosoma, with the integument pointed (Figs 3, 4). There are three pairs of coxal plates located on the proximal half of the idiosoma, and all of they are wider than long. The anterior coxa bears two setae, the medial coxa is without seta, and the posterior coxa has only one seta (Fig. 4). Gnathosomal sucker has a large disk, tibiotarsal claws two in number and the same size, weakly bent, five tibiotarsal spines, three of them roughly barbed (Figs 3, 4). The body sizes of the larvae of *H. processifera* (N = 3) are as follows in µm – idiosoma: L/W 290–300/230–233; dorsal shield: L/W 245–296/170–180; coxal plates: Cx-1 L/W 75–80/38–43, Cx-2 L/W 79–86/33–37, Cx-3 77–84/40–45; gnatosoma; L/W 200–203/148–150; pedipalpal segments: femur L/W 68–70/36–38, genu L 16–17, tibiotarsus L 58–61.

## Discussion

*Hydrachna inermis* was described from a site in Germany (Piersig 1895, 1897, 1899). Later it was recorded in various parts of Europe but without clearly defined diagnostic features; it was synonymized with *H. processifera* by Davids et al. (2005, 2007). *Hydrachna processifera* is the only species of genus *Hydrachna* which attaches to its hosts on the external integument; all other species attach the under elytra (Zawal 2002). In this study, all larvae of *H. processifera* were found on mesosternum. Zawal (2002) stated that the greatest numbers of larvae were found on the prosternum (40.3%), followed by the mesosternum (20.8%), and the least number on the metasternum (19.5%), and that they infested three species of *Dytiscus* (*D. circumcinctus, D. dimidiatus, D. marginalis*). For the three species of water beetles, *D. marginalis* was the least infected (prevalence = 0.7%). In the present study, the prevalence (16.7%) and intensity of infestation (11 individuals) of *D. marginalis* was higher than of *D. marginalis* in Zawal’s research and similar to prevalence and intensity of infestation of *D. circumcinctus* (Zawal 2002). Of course, the data obtained here should be approached with great caution, as they are based on a very small number of observations. The present study confirms a low prevalence and intensity of infestation of water beetles and water bugs found by other authors (Zawal 2002, 2003b, Biesiadka and Cichocka 1994) compared to dragonflies (Baker et al. 2008, Zawal and Szlauer-Łukaszewska 2012, Zawal and Buczyński 2013, Zawal et al. 2017) but similar to flies and caddisflies (Fairchild and Lewis 1987, Martin et al. 2010, Buczyńska et al. 2015, Stryjecki et al. 2015).

The small size all of water mite larvae of *H. processifera* confirms that the reproductive time is during summer. Wainstein (1980) noted that oviposition of this species takes place in July and developing requires 2–4 weeks in Russia. Zawal (2002) also reported greatest number of small and median sized larvae *H. processifera* in summer-autumn of Poland.

The water mite larvae collected from Muş Province were identified as *H. processifera* on the basis of shape of idiosoma and gnatosoma, and the shape of coxae and its setation. All tibiotarsal claws were large and of equal size. Unlike other larvae of water mites attached under elytra, larvae of water mites *H. processifera* are attached to outer surface of beetle bodies (Fig. 2). The record confirms the presence of this widely distributed, Palearctic species in the south-eastern part of its range (Turkey).

The redescribed of *H. processifera* is identical to Wainstein’s (1980) *H. inermis* larva in all features excluding length of pedipalpal femur and genu. The redescription add new features as: striated integument and pointed dorsal shield; and some measurements.
